# Impact of anionic polyacrylamide on stability and surface properties of the Al_2_O_3_–polymer solution system at different temperatures

**DOI:** 10.1007/s00396-016-3906-7

**Published:** 2016-07-09

**Authors:** Małgorzata Wiśniewska, Stanisław Chibowski, Teresa Urban, Dariusz Sternik, Konrad Terpiłowski

**Affiliations:** 1 Department of Radiochemistry and Colloid Chemistry, Faculty of Chemistry, Maria Curie-Sklodowska University, M. Curie-Sklodowska Sq. 3, 20-031 Lublin, Poland; 2 Department of Physicochemistry of Solid Surface, Faculty of Chemistry, Maria Curie-Sklodowska University, M. Curie-Sklodowska Sq. 3, 20-031 Lublin, Poland; 3 Department of Physical Chemistry—Interfacial Phenomena, Faculty of Chemistry, Maria Curie-Sklodowska University, M. Curie-Sklodowska Sq. 3, 20-031 Lublin, Poland

**Keywords:** Anionic PAM, Alumina surface, Thermogravimetric analysis, Temperature effect, Carboxyl groups content, Electrosteric stabilization

## Abstract

The stability mechanism and thermal properties of the system alumina–anionic polyacrylamide (PAM) was studied. The polymer’s adsorption properties in dependence on the following parameters solution pH (in the range 3–9), temperature (in the range 15–35 °C), and carboxyl groups’ content in the PAM chains (in the range 5–30 %) were examined. The turbidimetry method was applied for determination of the suspension stability of alumina in the presence of PAM. The obtained results indicate that the polymer addition improves significantly Al_2_O_3_ suspension stability at pH 6 and 9 (in the whole examined temperature range). PAM containing a larger number of carboxyl groups stabilizes solid particles more effectively (due to greater contribution of electrosteric interactions). Moreover, the polymer adsorption on the alumina surface causes changes in the thermal stability of the examined systems. In dependence on temperature, the higher the content of carboxyl groups in the PAM molecules, the greater the total mass loss. This is due to increased adsorption of polyacrylamide whose chains contain numerous –COOH groups.

**Electronic supplementary material:**

The online version of this article (doi:10.1007/s00396-016-3906-7) contains supplementary material, which is available to authorized users.

## Introduction

The adsorption process of different substances (simple ions, small molecules, surfactants, macromolecular compounds) takes place in many industrial and ecological operations. It can occur at various types of interfaces: gas–liquid, gas–solid, liquid–solid, or liquid–liquid. Adsorption in a solid–liquid system differs substantially from this process in a gas–solid system. This results from the fact that in the former case, the solution is adsorbate (i.e., in the simplest system—binary mixture of solvent and solute). The competition between the two components of the solution for active sites on the surface occurs. The adsorption process at gas–solid and liquid–solid interfaces finds widespread applications in the environmental protection. Different kinds of adsorbents are used in order to reduce atmospheric emissions of harmful gases and vapors [1–4], as well as for removal of poisonous chemical compounds dissolved or dispersed in wastewaters [5–12].

The ability of polymers to modify surface properties of solids promoted their use in stabilization or destabilization of colloidal particles. For this reason, they are widely used in many fields of industry, technology, medicine, and ecology [13–17]. Nevertheless, adsorption of the polymer on the solid surface is a very complex process which differs significantly from the adsorption of small molecules [18]. Macromolecules binding with the surface of the adsorbent particles are associated with the decrease in free energy of components in the system. This involves multipoint connections of the polymer functional groups to the solid surface. However, not all polymer segments are directly bound to the solid surface. The polymer adsorption process is possible when the system exceeds the critical adsorption energy value. In such a case, entropy effects counteract attraction forces between the polymer and the surface.

The polymer adsorption kinetics is determined in the following stages of this process: (a) polymeric chains transport toward the solid surface (depending on the convection and diffusion processes), (b) rate of polymer binding to the adsorbent surface (depending on the height of activation energy barrier), (c) reconformation of adsorbed macromolecules which minimizes their free energy, and (d) desorption of shorter polymer chains by longer ones (for polydisperse polymers) [19]. Each of these stages is specific to a given polymer–solid system. Accordingly, the polymer adsorption depends, among others, on the molecular weight of the polymer and its distribution (polydispersity), initial polymer concentration, pH and ionic strength of the solution, temperature, solvent type, as well as mixing degree of the individual components of the system.

The addition of the polymer to a colloidal suspension may result in its stabilization or destabilization [20]. The total coverage of the particle surface by polymer film (usually at a high polymer concentration) results in the steric stabilization. It involves the presence of two effects. The first one—entropic—refers to the reduction of the macromolecule conformational entropy by decrease of the surface area available for polymer segments. The second one—mixing—is associated with the increase of polymeric segment concentration in the area of the mutual penetration of polymer adsorption layers and changes in osmotic pressure. The particles whose surfaces are covered by the adsorbed polymer repel due to the Gibbs energy increase. When the stabilizing polymer is a polyelectrolyte, the electrostatic repulsion between polymeric layers (possessing the same charge) can also occur.

When a small amount of polymer (that does not guarantee complete coverage of the surface) is added to the dispersion, one polymer chain can adsorb onto two or more colloidal particles forming polymer bridges between them. These bridges are formed when the length of loops and tails of the adsorbed macromolecules is greater than the range of electrostatic repulsions between the colloidal particles. As a result, flocks undergo sedimentation and the system destabilization occurs.

Polymers which do not adsorb on the solid surface (high affinity of the polymer for the solvent, complete coverage of the colloidal particles by the surfactant molecules) cause depletion stabilization or flocculation.

The main aim of this study was the determination of the stability properties of anionic polyacrylamide (PAM; with a differing content of carboxyl groups) in the aqueous suspension of aluminum(III) oxide in the temperature range 15–35 °C. Thermogravimetric analysis of the examined systems was also performed to obtain the additional information about PAM adsorption mechanism. The temperature impact on the conformation of adsorbed macromolecules is significant [21–23] due to modification of interactions between the polymeric chains and the solvent molecules. The ability to influence the structure of polymeric adsorption layer by temperature change is closely related to the suspension stability. Nevertheless, this problem is marginally described in the scientific literature [24–26]. Therefore, the presented studies can supplement incomplete knowledge of this topic.

## Materials and methods

Aluminum(III) oxide—Al_2_O_3_ (Merck)—with the specific surface area 155 m^2^/g was used as an adsorbent. This metal oxide was washed with doubly distilled water to achieve the supernatant conductivity below 2 mS/cm. The mean particle diameter of the solid was 469 nm (Zetasaizer 3000, Malvern Instruments). High surface area, minimal solubility, and high mechanical strength promoted to Al_2_O_3_ use in the experiments.

Anionic PAM (Korona) was applied as an adsorbate. Polymer samples differed in contents of carboxyl groups (5, 20, and 30 %). These anionic groups remained in macromolecules as a result of incomplete hydrolysis of a number of the amide groups during the PAM preparation. Carboxyl groups underwent dissociation with the increasing pH value and are a source of negative charge of the polyacrylamide chains [27, 28]. The characteristics of the polyacrylamide samples are listed in Table 1. p*K*a values of PAM were determined using the potentiometric titration method. Knowing the p*K*a value, calculation of dissociation degree (*α*) of the polymer carboxyl groups was possible. Table 1 presents the values of these parameters. At pH 3, 16.6 % of anionic groups are ionized, whereas at the pH values 6 and 9, the dissociation is practically complete.

**Table 1:** PAM probe characteristics

Molecular weight/Da	Carboxyl group content/%	Symbol	p*K*a	*α*/% pH 3
11,000,000	5	11_5%	3.7	16.6
14,000,000	20	14_20%	3.7	16.6
14,000,000	30	14_30%	3.7	16.6

All measurements were carried under the following conditions: pH range 3–10, temperature range 15–35 °C, and supporting electrolyte—NaCl with the concentration 0.01 mol/dm^3^.

Stability measurements of the alumina suspensions (without and with PAM) were carried out with Turbiscan Lab^Expert^ with the cooling module TLAb Cooler (France). This apparatus registers light (with the initial wavelength 880 nm) passing through both the examined system and scattered by the solid particles dispersed in the liquid medium. The computer programs (TLab EXPERT 1.13 and Turbiscan Easy Soft) working with a turbidimeter present the obtained data in the form of transmission and backscattering curves (so-called scans). On the *y* axis, the intensity of transmission (or backscattering) is marked, whereas on the *x* axis, the suspension level in the measurement vial is shown. The suspension was added into the glass vial (70 mm long) to about 40 mm of its height. Changes in the suspension stability were monitored for 15 h, and single scans were collected every 15 min (appropriate colors of scans correspond to particular times of the experiment).

The backscattering data are also used for calculation of the stability coefficient Turbiscan stability index (TSI) according to the equation:

1 $$ TSI=\sqrt{\frac{{\displaystyle \sum_{i=1}^n{\left({x}_i-{x}_{BS}\right)}^2}}{n-1}} $$

where *x*_*i*_ is the average backscattering for each minute of measurement, *x*_BS_ is the average *x*_1_, and *n* is the number of scans.

The suspension with 0.02 g of aluminum(III) oxide in 20 cm^3^ of NaCl solution was sonicated for 1 min (Sonics, LABO PLUS). Then, the required pH of the solution was adjusted (3, 6, or 9 ± 0.1) using pH Φ360 pH/Temperature/mV Meter (Beckman). The suspension was shaken in a water bath (OLS 200, Grant) for 30 min, and during this time, its pH was checked. The probes of the alumina suspension containing polyacrylamide were prepared in a similar way. An appropriate volume of the stock PAM solution (with the concentration 1100 ppm), providing its final concentration 100 ppm, was added to the suspension directly before stability test. After that, the system pH was checked and the measuring vial with the suspension was immediately placed in the thermostated chamber of turbidimeter.

Adsorption experiments were made by the static method in the polymer concentration range 5–120 ppm at the pH values 3, 6, and 9 (±0.1) using 0.05 g of Al_2_O_3_. Such prepared suspensions were shaken in the water bath OLS 200 Grant for 24 h. After that, the solids were centrifuged using a microcentrifuge (type MPW-223e, MPW Med Instruments). The reaction of polyacrylamide with hyamine proposed by Crummet and Hummel [28] was applied. The solution turbidity was measured after 15 min using the ultraviolet-visible (UV-VIS) spectrophotometer (Carry 1000; Varian) at 500 nm. To determine the amount of adsorbed polyacrylamide, the difference between the initial PAM concentration and that after the adsorption process was calculated (using the calibration curve obtained earlier).

The probes for thermal analysis were prepared by adding 0.1 g of Al_2_O_3_ to 25 cm^3^ of NaCl or NaCl with PAM (*C*_PAM_ = 200 ppm) solutions. Due to the fact that anionic PAM shows the greatest adsorption on the alumina surface at pH 3, this value of pH was adjusted in the examined suspensions. Then, they were shaken in a water bath for 24 h, and meanwhile, their solution pH was checked. After this time, these probes were centrifuged and the solid (with or without the polymer) was dried.

Thermal analysis was carried out on a STA 449 Jupiter F1, Netzsch (Germany) under the following operational conditions: heating rate of 10 °C/min, a dynamic atmosphere of synthetic air (50 mL/min), temperature range of 30–950 °C, sample mass ~25 mg, and sensor thermocouple type S TG-DSC. As a reference, empty Al_2_O_3_ crucible was used. The gaseous products emitted during decomposition of the materials were analyzed by QMS 403C Aeölos (Germany) coupled online to the STA instrument. The QMS data were gathered in the range from 10 to 300 amu.

## Results and discussion

The calculated values of TSI stability coefficients are listed in Table 2. Their analysis requires knowledge of the fact that TSI assumes values in the range 0–100. Small TSI values indicate high stability of the examined suspension, whereas increase in the TSI value corresponds to deterioration of system stability conditions. As can be seen, alumina suspension without the polymer is successively unstable (for both examined temperatures)—TSI values change in the range 33–63 (with the exception of the system at pH 6 and 15 °C). Generally, the anionic polyacrylamide addition improves stability of the dispersed solid particles (at fixed pH and temperature, the decrease of TSI values for the system containing PAM in relation to that without polymer is observed).

**Table 2:** Stability coefficient TSI for all examined systems, *C*_PAM_ = 100 ppm

Al_2_O_3_ suspension	Temperature/°C	TSI		
		pH 3	pH 6	pH 9
Without PAM	15	33.65	19.91	54.23
	35	38.75	55.14	63.45
With PAM 11_5%	15	20.93	16.71	17.09
	35	35.14	29.97	47.20
With PAM 14_20%	15	28.91	8.88	9.48
	35	35.1	20.72	12.47
With PAM 14_30%	15	23.12	3.89	8.97
	35	31.57	19.29	18.59

**Fig. 1:**
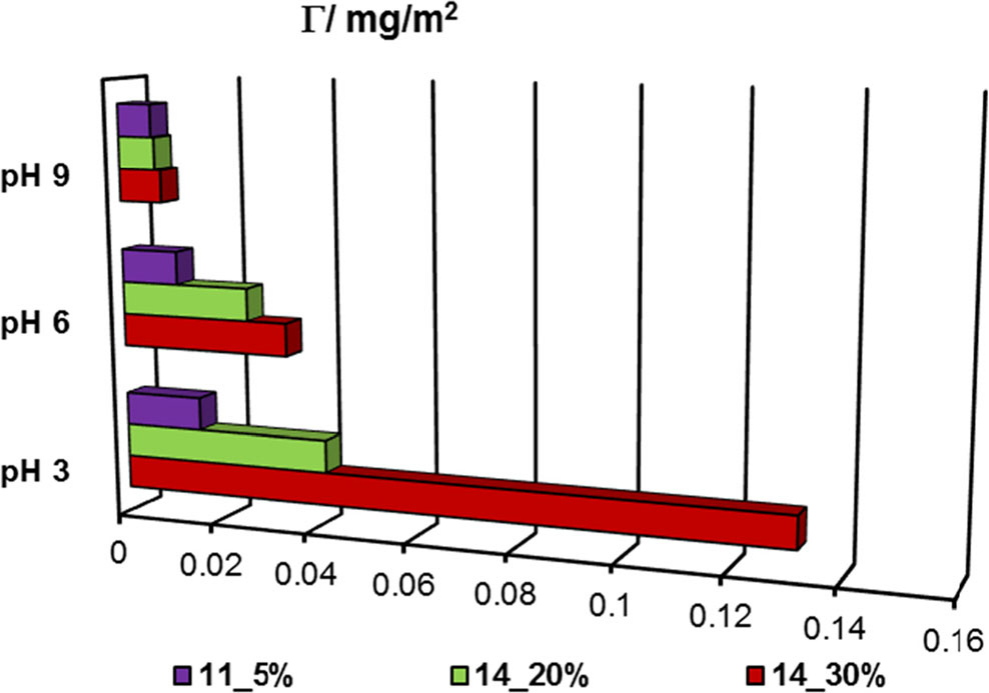
Adsorbed amounts of PAM on the alumina surface at 15 °C for different solution pH values and anionic group content in polymer chains; *C*_PAM_ = 100 ppm

**Fig. 2:**
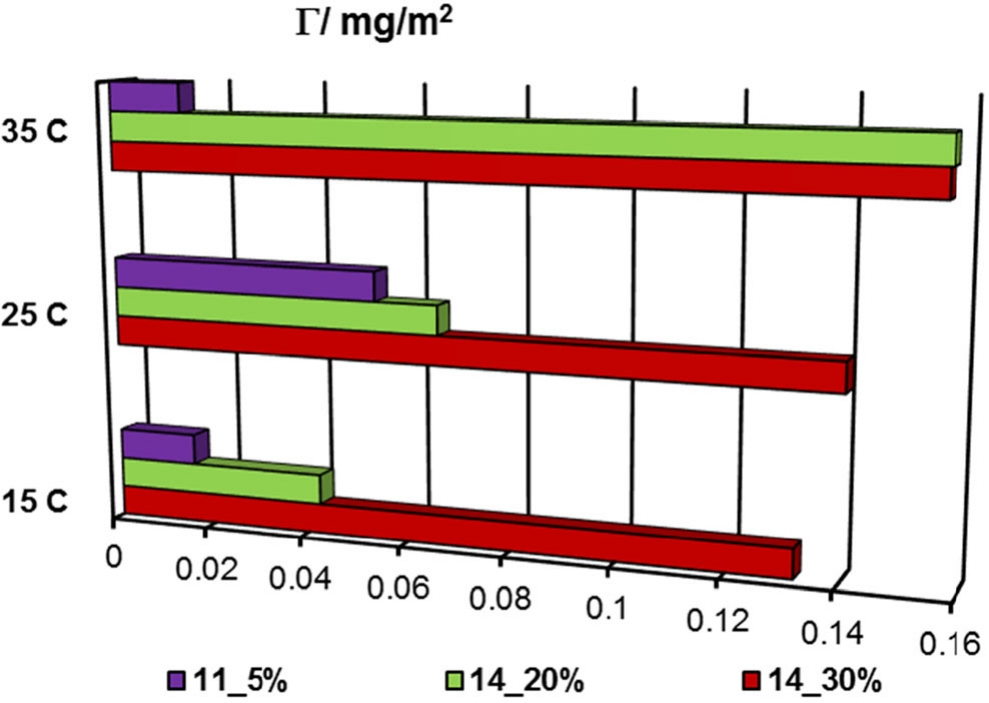
Adsorbed amounts of PAM on the alumina surface at pH 3 for different temperature values and anionic group content in polymer chains; *C*_PAM_ = 100 ppm

To explain stability changes of the alumina particles dispersed in the polyacrylamide solution, the information about polymer adsorption is necessary. These data are discussed in detail in our previous paper [29], and in the present manuscript, a few relevant results are presented in Figs. 1 and 2. Their analysis indicates that adsorption of anionic PAM on the Al_2_O_3_ surface increases with the rise of temperature (besides PAM 11_5%) and carboxyl group content in macromolecules, whereas its decrease is observed with the increasing pH.

Solution pH affects both dissociation of the PAM carboxyl groups and alumina surface charge. The pH_pzc_ (point of zero charge (pzc)) of Al_2_O_3_ in the NaCl solution changes in the range 7.46–8.07 (in the temperature range 15–35 °C) [29]. The greatest adsorption of anionic PAM at pH 3 is a result of electrostatic attraction between the positively charged solid surface and the minimally negatively charged PAM macromolecules (which assumes more coiled conformation assuring their dense packing on the solid surface). The formation of hydrogen bonds between the polymer functional groups (both carboxyl and amide) and the solid surface sites is also possible [30]. The total dissociation of polymer anionic groups at pH 6 and 9 leads to development of polymeric chains, which occupy a larger surface area (adsorption decrease at pH 6) or repulse electrostatically with the negatively charged metal oxide surface (the lowest adsorption level at pH 9).

The temperature increase promotes more extended conformation of polymer chains (increase of hydrodynamic radius of polymer coil) [22]. At a fixed pH value and at higher examined temperature, a thicker adsorption layer, composed of macromolecules expanded perpendicularly to the alumina surface, was formed. As a consequence, the greater number of polymeric molecules can adsorb on the solid surface area unit (adsorption increase).

Only in the case of PAM 11.0_5% (whose chains contain the smallest amount of carboxyl groups), the highest adsorption level was observed at 25 °C. Conformation changes of its macromolecules due to temperature increase are not enhanced so effectively by a negatively charged carboxyl groups in polymer chains as is in the case of other polymer samples (with a higher content of anionic groups).

The polyacrylamide-adsorbed amount increase with the rise of carboxyl group content is also observed. More numerous anionic groups cause more expanded conformation of polymeric chains. Such structure of adsorbing macromolecules favors their higher adsorption in the alumina–polymer solution system.

Based on the turbidimetric and adsorption results, a more probable mechanism of suspension stability in the polymer presence can be proposed.

At pH 3, there is a slight influence of PAM adsorption on the Al_2_O_3_ suspension stability (for both examined temperatures). It is probably caused by the greatest polymer adsorption and formation of a densely packed polyacrylamide layer on the colloidal particle surfaces. This results in the appearance of steric repulsion between the solid particles which leads to slight improvement of the solid suspension stability in the PAM presence.

Improvement of stability of the alumina suspension in the presence of polyacrylamide is more pronounced at two other pH values. TSI coefficient for the samples containing PAM assumes significantly lower values (compared to the oxide suspension without the polymer). The greater the improvement of suspension stability is, the higher content of carboxyl groups in the polyacrylamide chains is found.

At pH 6, the adsorbed macromolecules assume a more extended conformation. Despite less adsorption of the polymer (in comparison to that at pH 3), the alumina suspension stability increases due to the rise of repulsive interactions between the solid particles covered with polymeric layers. The mechanism of stabilization is electrosteric.

A similar effect occurs in the systems at pH 9 at which the adsorption of PAM is significantly lower (electrosteric forces between alumina particles). Because of low adsorption of the polymer, some depletion interactions (caused by unadsorbed PAM chains) can be of importance.

**Fig. 3:**
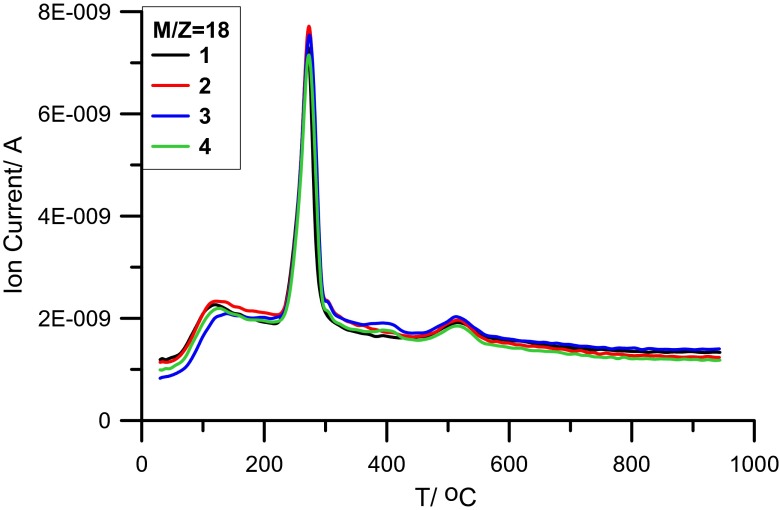
MS profile of H_2_O vs temperature for systems: Al_2_O_3_ (*1*), Al_2_O_3_/PAM 11_5% (*2*), Al_2_O_3_/PAM 14_20% (*3*), and Al_2_O_3_/PAM 14_30% (*4*)

**Fig. 4:**
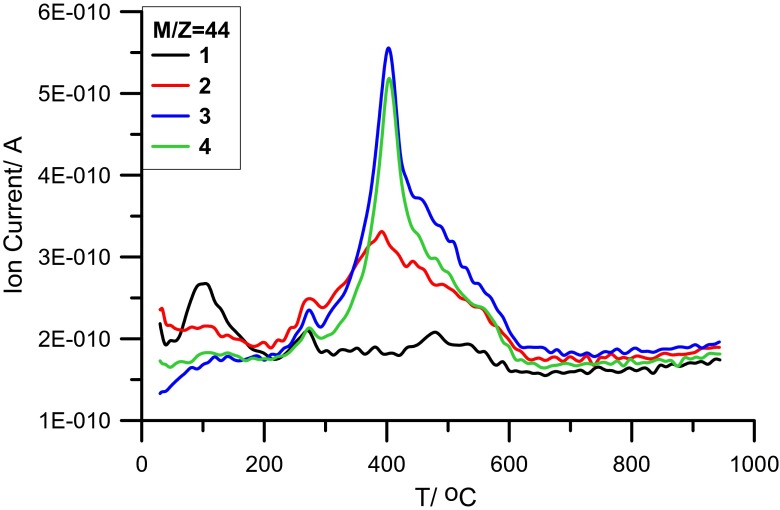
MS profile of CO_2_ vs temperature for systems: Al_2_O_3_ (*1*), Al_2_O_3_/PAM 11_5% (*2*), Al_2_O_3_/PAM 14_20% (*3*), and Al_2_O_3_/PAM 14_30% (*4*)

For detailed analysis of processes taking place during the thermal degradation of the examined samples, measurements of the presence of gaseous products by mass spectrometry were performed. The intensity profiles of main decomposition products (H_2_O, CO_2_) are presented in Figs. 3 and 4. The analysis of *m*/*z* = 18 (characteristic of H_2_O) indicates small changes obtained for the alumina samples modified by PAM in relation to the solid sample without the polymer. This demonstrates that the binding process of polymer with solid is of the surface character. This behavior is confirmed by slight differences in enthalpy values calculated from the peak profiles on the DSC curves (Fig. 10, Supplementary Information section)—for desorption of physically (Δ*H*_I_) and chemically (Δ*H*_II_) bound water (Table 3). Only in the temperature range 350–450 °C, the appearance of additional peak is observed [31–37]. The presence of this peak is associated with the organic hydrogen oxidation.

**Table 3:** The heat values of main stages of thermal degradation of samples determined by DSC analysis

Sample	Δ*H*_I_/J/g	Δ*H*_II_/J/g	Δ*H*_III_/J/g
Al_2_O_3_	16.37	64.15	–
Al_2_O_3_/PAM 11_5%	18.97	62.42	−1.12
Al_2_O_3_/PAM 14_20%	18.79	63.04	−18.9
Al_2_O_3_/PAM 14_30%	16.32	61.68	−16.2

In the case of *m*/*z* = 44 (characteristic of CO_2_), the appearance of distinct peaks in the temperature range 250–600 °C with the maximum at about 400 °C was observed. This is an evidence of organic substance decomposition. The obtained effects depend mainly on the adsorbate molecule structure and its adsorbed amount (changes in Δ*H*_III_ values).

**Fig. 5:**
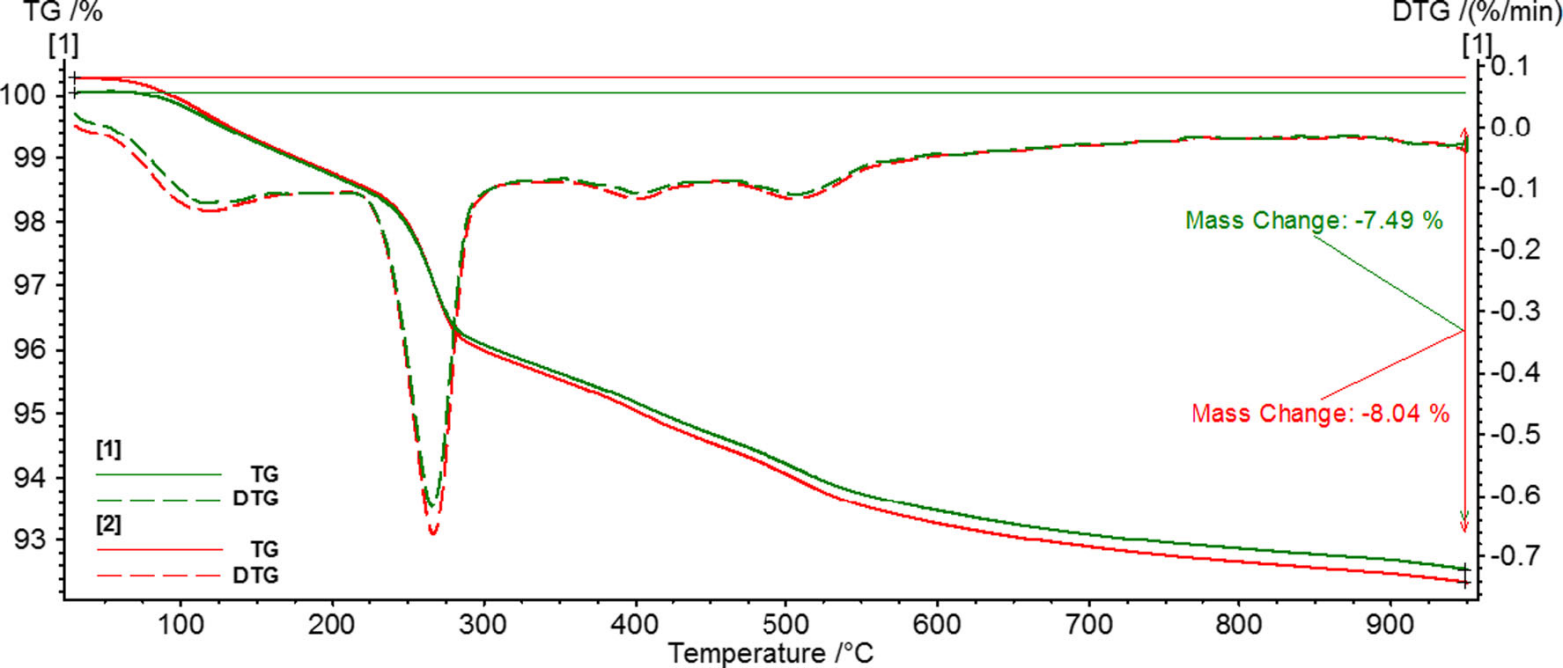
TG and DTG curves for alumina systems modified by PAM 14_30% at pH 3: adsorbed at 15 °C (*1*) and adsorbed at 35 °C (*2*)

The influence of temperature, at which alumina surface modification with anionic PAM was performed, on the thermal characteristics of examined systems is shown in Fig. 5. It represents the TG and derivative thermogravimetric (DTG) curves obtained for alumina with adsorbed PAM 14_30% at 15 and 35 °C. The analysis of these curves indicates that the total mass change is greater in the case of the sample prepared at 35 °C. This is a result of greater adsorption level of PAM obtained at the highest examined temperature.

## Conclusions

Adsorption of anionic PAM on the surface of aluminum(III) oxide decreases with the rising pH. At pH 3, there is a small dissociation of PAM carboxyl groups, and therefore, the adsorption proceeds by hydrogen bond formation and slight electrostatic attraction. At pH 3, there is a slight influence of PAM adsorption on the Al_2_O_3_ suspension stability (for both examined temperatures).

Stronger electrostatic attraction between the negatively charged macromolecules and the positively charged Al_2_O_3_ surface is mainly responsible for the polymer adsorption at pH 6. Despite less adsorption of the polymer (in comparison to that at pH 3), the alumina suspension stability increases due to the rise of repulsive interactions between the solid particles covered with polymeric layers (electrosteric stabilization).

The smallest polymer adsorption at pH 9 is a result of repulsion between the PAM chains and Al_2_O_3_ particles (both negatively charged). Because of low adsorption of the polymer, some importance of suspension stability improvement can have depletion interactions (caused by unadsorbed PAM chains).

The higher the content of anionic carboxyl groups is in macromolecules and temperature, the larger the amount of adsorbed polyelectrolyte is. This is due to the conformational changes of macromolecules manifested by adopting a more developed structure in the adsorption layer.

The adsorption of anionic PAM causes noticeable decrease in thermal stability of the alumina. The greater the total mass losses are, the higher the polymer adsorption is (i.e., the higher is the temperature of this process and is carboxyl groups’ content in the PAM chains). For the alumina samples with the polymer, the additional minimum on the DTG curves appears at about 402 °C (thermo-oxidation of the organic H atoms and carbonized polymer residue).

## Electronic supplementary material

ESM 1 (DOCX 398 kb)

## References

[1] Nowicki P, Pietrzak R, Wachowska H (2008). Comparison of physicochemical properties of nitrogen-enriched activated carbons prepared by physical and chemical activation of brown coal. Energy Fuel.

[2] Nowicki P, Pietrzak R, Wachowska H (2009). Influence of metamorphism degree of the precursor on preparation of nitrogen enriched activated carbons by ammoxidation and chemical activation of coals. Energy Fuel.

[3] Nowicki P, Pietrzak R (2011). Effect of ammoxidation of activated carbons obtained from sub-bituminous coal on their NO_2_ sorption capacity under dry conditions. Chem Eng J.

[4] Nowicki P, Supłat M, Przepiórski J, Pietrzak R (2012). NO_2_ removal on adsorbents obtained by pyrolysis and physical activation of corrugated cardboard. Chem Eng J.

[5] Nosal-Wiercińska A, Grochowski M, Wiśniewska M, Tyszczuk-Rotko K, Skrzypek S, Brycht M, Guziejewski D (2015). The influence of protonation on the electroreduction of Bi(III) ions in chlorates (VII) solutions of different water activity. Electrocatalysis.

[6] Ostolska I, Wiśniewska M (2015). The polymer structure impact on adsorption of the ionic polyamino acid homopolymers and their diblock copolymers on the colloidal chromium (III) oxide. RSC Adv.

[7] Wiśniewska M, Chibowski S, Urban T (2015). Impact of polyacrylamide with different contents of carboxyl groups on the chromium (III) oxide adsorption properties in aqueous solution. J Hazard Mater.

[8] Nowicki P (2016). The effect of mineral matter on the physicochemical and sorption properties of brown coal-based activated carbons. Adsorption.

[9] Gupta VK, Pathania D, Agarwal S, Sharma S (2012). Removal of Cr (VI) onto *Ficus carica* biosorbent from water. Environ Sci Pollut Res.

[10] Gupta VK, Ali I, Saini VK (2007). Defluoridation of wastewaters using waste carbon slurry. Water Res.

[11] Nosal-Wiercińska A (2012). Adsorption of cystine at mercury/aqueous solution of chlorate (VII) interface in solutions of different water activity. Cent Eur J Chem.

[12] Nosal-Wiercińska A, Grochowski M (2011). Adsorption of thiourea and its methyl derivatives from chlorate(VII) with varied water activity. Collect Czechoslov Chem Commun.

[13] Novio F, Simmchen J, Vazquez-Mera N, Amorin-Ferre L, Ruiz-Molina D (2013). Coordination polymer nanoparticles in medicine. Coord Chem Rev.

[14] Allen TM, Cullis PR (2013). Liposomal drug delivery systems: from concept to clinical applications. Adv Drug Deliv Rev.

[15] Bajpai AK, Shukla SK, Bhanu S, Kankane S (2008). Responsive polymers in controlled drug delivery. Prog Polym Sci.

[16] Brostow W, Hagg Lobland HE, Pal S, Singh RP (2009). Polymeric flocculants for wastewater and industrial effluent treatment. J Mater Educ.

[17] Chen HT, Ravishankar SA, Farinato RS (2003). Rational polymer design for solid–liquid separations in mineral processing applications. Int J Miner Process.

[18] Adamson AW (1997). Physical chemistry of surfaces.

[19] Fleer GJ, Cohen Stuart MA, Scheutjens JMHM, Cosgrove T, Vincent B (1993). Polymers at interfaces.

[20] Napper DH (1983). Polymeric stabilization of colloidal dispersions.

[21] Wiśniewska M (2010). Temperature effect on adsorption properties of silica–polyacrylic acid interface. J Therm Anal Calorim.

[22] Wiśniewska M (2012). The temperature effect on the adsorption mechanism of polyacrylamide on the silica surface and its stability. Appl Surf Sci.

[23] Wiśniewska M (2012). Temperature effects on the adsorption polyvinyl alcohol on silica. Cent Eu J Chem.

[24] Guo LC, Zhang Y, Uchida N, Uematsu K (1997). Influence of temperature on stability of aqueous alumina slurry containing polyelectrolyte dispersant. J Eur Ceram Soc.

[25] Mpofu P, Addai-Mensah J, Ralston J (2004). Temperature influence of nonionic polyethylene oxide and anionic polyacrylamide on flocculation and dewatering behavior of kaolinite dispersions. J Colloid Interface Sci.

[26] O’Shea JP, Qiao GG, Franks GV (2010). Solid-liquid separations with a temperature-responsive polymeric flocculant: effect of temperature and molecular weight on polymer adsorption and deposition. J Colloid Interface Sci.

[27] Wiśniewska M, Chibowski S, Urban T (2015). Modification of the alumina surface properties by adsorbed anionic polyacrylamide—impact of polymer hydrolysis. J Ind Eng Chem.

[28] Crummett WB, Hummel RA (1963). The determination of traces of polyacrylamides in water. J Am Water Works Assoc.

[29] Wiśniewska M, Chibowski S, Urban T (2016). Influence of temperature on adsorption mechanism of anionic polyacrylamide in the Al_2_O_3_–aqueous solution system. Fluid Phase Equilib.

[30] Kasprzyk-Hordern B (2004). Chemistry of alumina, reactions in aqueous solution and its application in water treatment. Adv Colloid Interf Sci.

[31] Sternik D, Staszczuk P, Sobieszek J, Płanda-Czyż M, Wasak S (2006). Influence of albumin adsorption on physico-chemical properties of alumina surfaces. J Therm Anal Calorim.

[32] Wiśniewska M, Chibowski S, Urban T, Sternik D (2011). Investigation of the alumina properties with adsorbed polyvinyl alcohol. J Therm Anal Calorim.

[33] Wiśniewska M, Chibowski S, Urban T (2013). Investigations of flocculation possibilities of the water alumina suspension in the presence of nonionic polymer. J Ind Eng Chem.

[34] Yariv S (2004). The role of charcoal on DTA curves of organo-clay complexes: an overview. Appl Clay Sci.

[35] Landau A, Zaban A, Lapides I, Yariv S (2002). Montmorillonite treated with rhodamine-6G mechanochemically and in aqueous suspensions. J Therm Anal Calorim.

[36] Sternik D, Majdan M, Deryło-Marczewska A, Żukociński G, Gładysz-Płaska A, Gun’ko VM, Mikhalovsky SV (2011). Influence of basic red 1 dye adsorption on thermal stability of Na-clinoptilolite and Na-bentonite. J Therm Anal Calorim.

[37] Majdan M, Pikus S, Gajowiak A, Sternik D, Zięba E (2010). Uranium sorption on bentonite modified by octadecyltrimethylammonium bromide. J Hazard Mater.

