# Supplementary material for: Impact of anionic polyacrylamide on stability and surface properties of the Al2O3–polymer solution system at different temperatures
Source: Colloid Polym Sci. 2016 Jul 9;294:1511–7. doi: 10.1007/s00396-016-3906-7 (PMC4978783; doi:10.1007/s00396-016-3906-7)
Supplement: Supplementary file 1 — (DOCX 398 kb) [file 396_2016_3906_MOESM1_ESM.docx]

**Supplementary information**

Some information about the suspension stability can be obtained also from the analysis of the course of transmission and backscattering curves. The exemplary data are presented in Figures 6 and 7. They correspond to the most stable system of all examined - Al_2_O_3_‑PAM 14_30% at pH 6 and 15 ^o^C (TSI = 3.89, Fig. 6) and the most unstable one - Al_2_O_3_ without PAM at pH 9 and 35 ^o^C (TSI = 63.45, Fig. 2).

As can be seen, for the suspension characterized by high stability (Fig. 6), the level of light transmission through such dispersion is 0% to the 35 mm height of the measuring vial. Above this height, the rapid increase of transmission to 50% occurs. The transmission peak presence indicates a clarification process of the suspension, but only in its top layer. The width of this peak corresponds to thickness of the clear layer. Moreover, the transmission curves obtained for individual time intervals overlap. It means that the stability conditions practically do not change in the total time of experiment (i.e. 15 hours). Simultaneously, the backscattering level changes in the range from 10% (for the top suspension layer) to 90% (for the peak indicating the thin sediment layer on the vial bottom).

On the other hand, for the most unstable system of all tested ones (Fig. 7), the transmission level reaches 50-70% at the 5-38 mm height of the measuring vial. The transmission is zero at the 0-5 mm height but at the same time at such height the backscattering peak is present. It indicates the presence of about 5 mm thick sediment layer. Additionally, with the exception of the bottom, the backscattering level is rather low (about 10%). The transmission level increases in the total time of experiment which suggests deterioration of the suspension stability with time.

Figures 8-10 present the TG, DTG and DSC curves for the alumina samples, both unmodified and modified with PAM (polymer adsorption conditions: pH 3 and 35^o^C). Under these pH and temperature conditions anionic polyacrylamide shows the greatest adsorption on the alumina surface.

One can distinguish three regions on the DTG curve (Fig. 9) of alumina without polymer [31-33]. The first weight loss of 1.73% in a low-temperature region (<200^o^C) with a minimum at about 106.7 ^o^C, is due to removal of physically adsorbed water. The second peak from 200 to 300^o^C with minimum at 264.8^o^C is associated with the endothermic thermodesorption of chemically adsorbed water. In the third a high-temperature range (>300^o^C with a minimum at 509.5^o^C) the weight loss of 2.93% corresponds to dehydroxylation.

The adsorption of anionic polyacrylamide causes noticeable changes in thermal characteristics of the alumina. These changes depend on anionic groups content in the polymeric chains. As can be seen in Fig. 8, the highest is the percentage content of carboxyl groups in PAM macromolecules, the greatest total mass losses are obtained. This is probably a result of larger adsorption of polyacrylamide whose chains contain more numerous –COOH groups. Moreover, for the alumina samples with polymer, four regions are present on the DTG curves (in the case of alumina without PAM three regions are observed). The additional minimum appears at about 402^o^C.

Similar changes were observed in the temperature range 250-700^o^C in the case of thermal decomposition of other organic compounds. At lower temperatures the partial oxidation of the H atoms of organic substance takes place, whereas at higher temperatures thermo-oxidation of H atoms and carbonized residue occurs [34,35].

The polymer adsorption has an impact on the thermal stability of solids. In the case of examined systems the decrease of total thermal stability of alumina with adsorbed PAM is observed (in relation to the solid sample without polymer), for which the dehydroxylation process proceeds at lower temperature. Similar changes, although much more intensive were obtained for the adsorption of surfactants and dyes on various types of materials [36,37]. This is also confirmed by the changes in temperature values corresponding to desorption of physically and chemically bound water (Fig. 9).


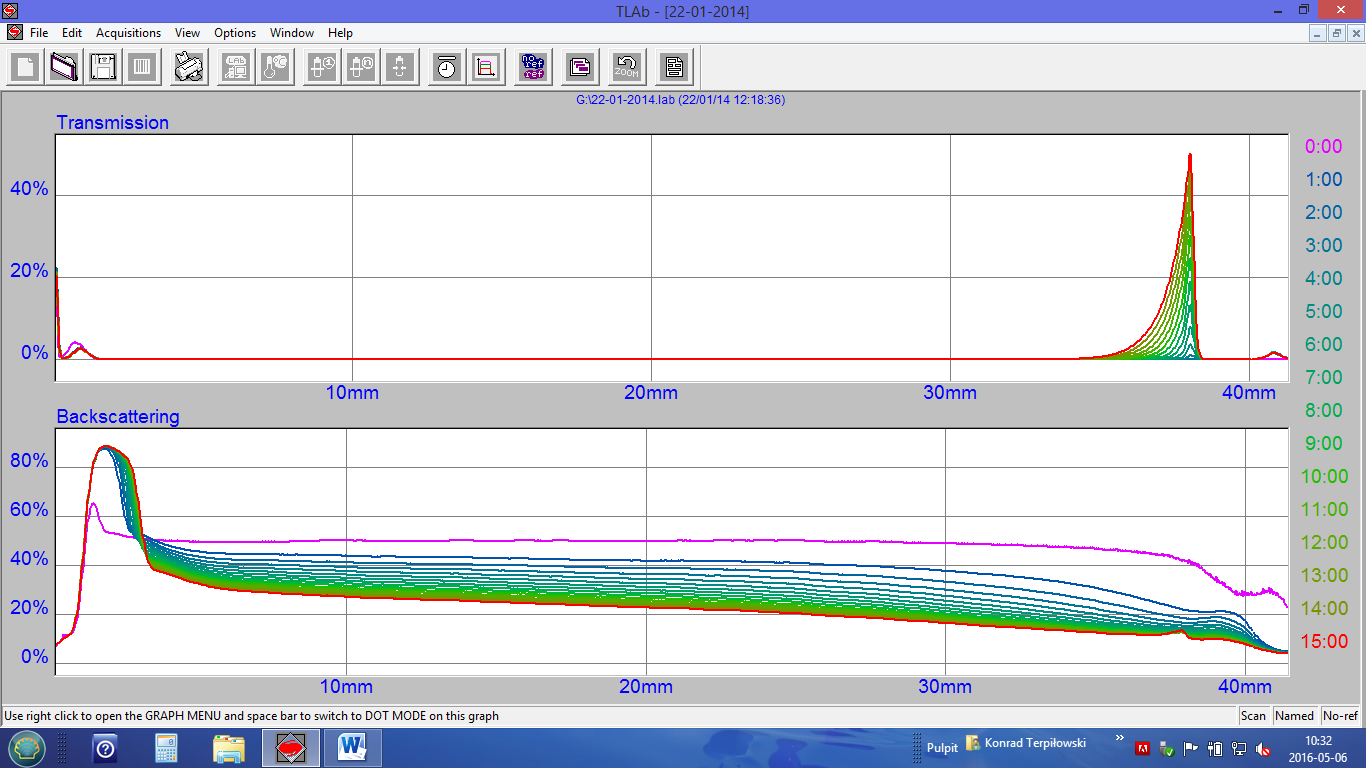


**Fig. 6.**


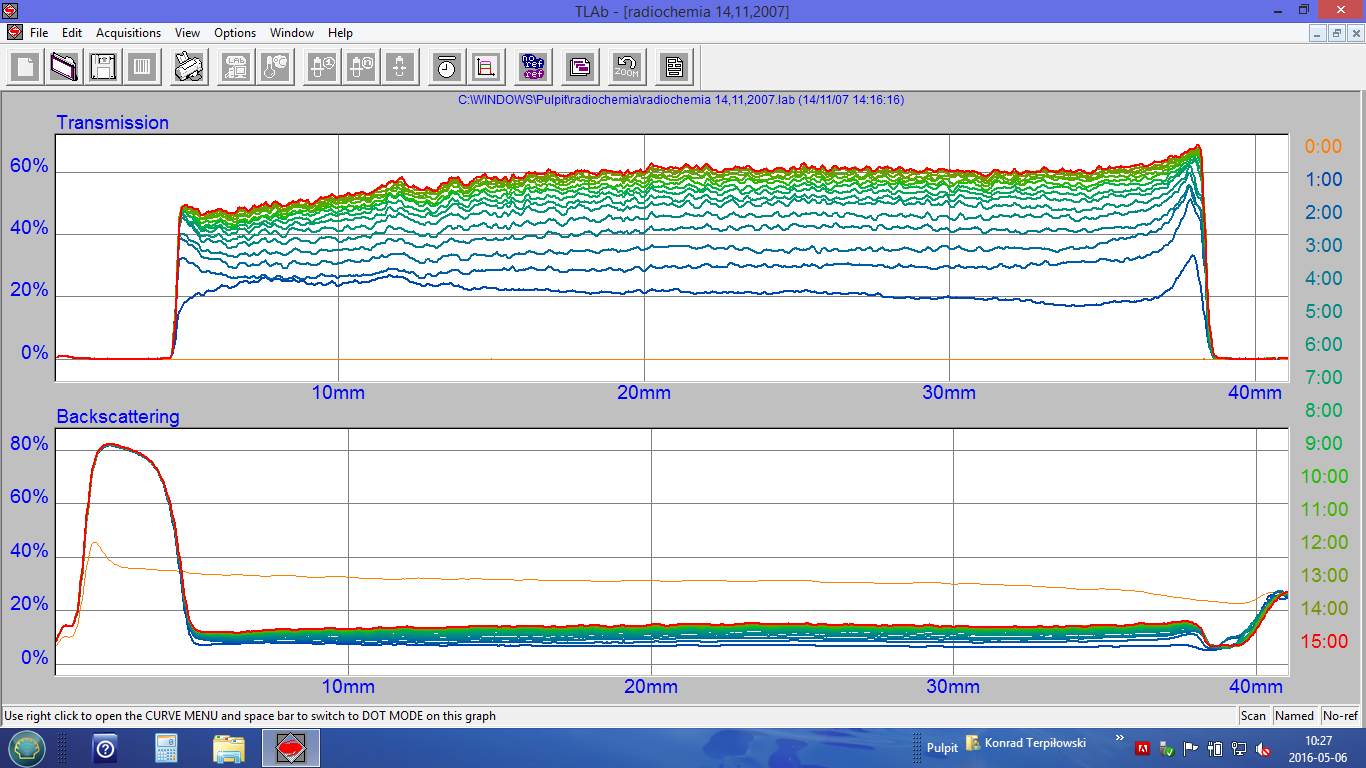


**Fig. 7.**


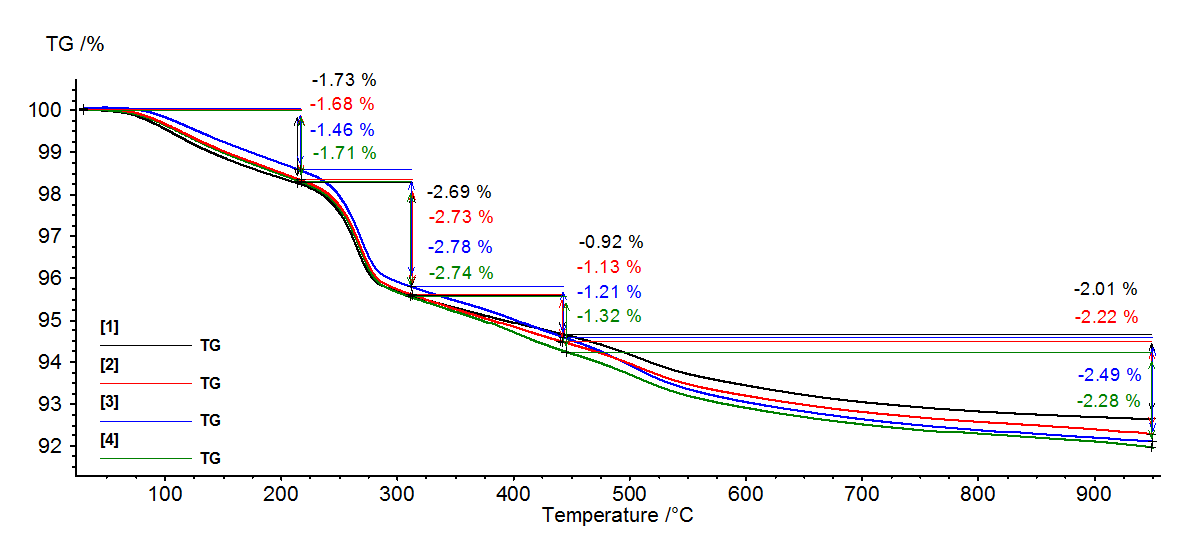


**Fig. 8.**

**
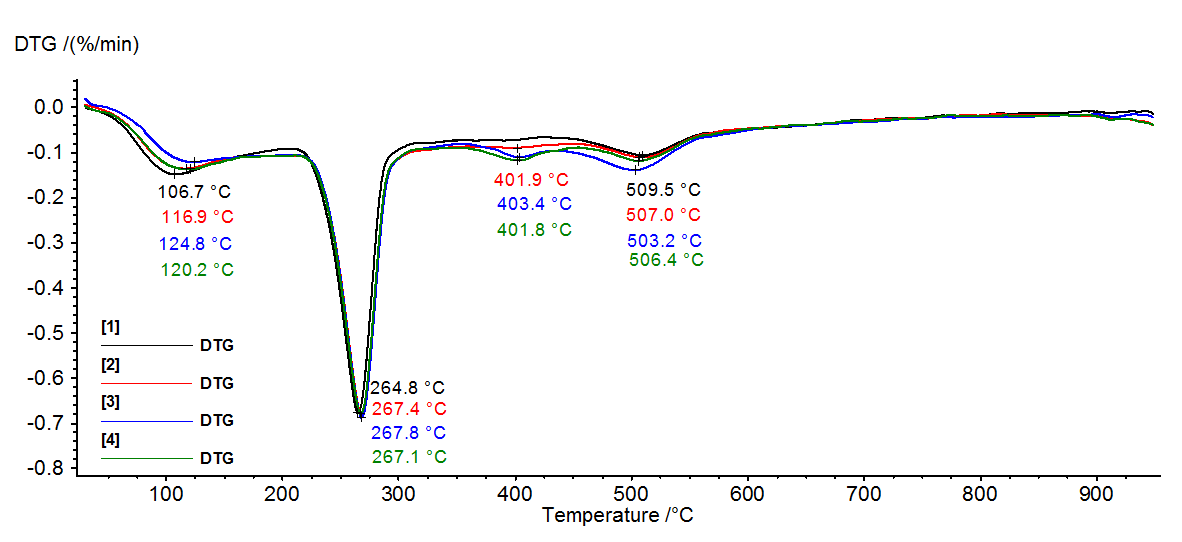
**

**Fig. 9.**

**
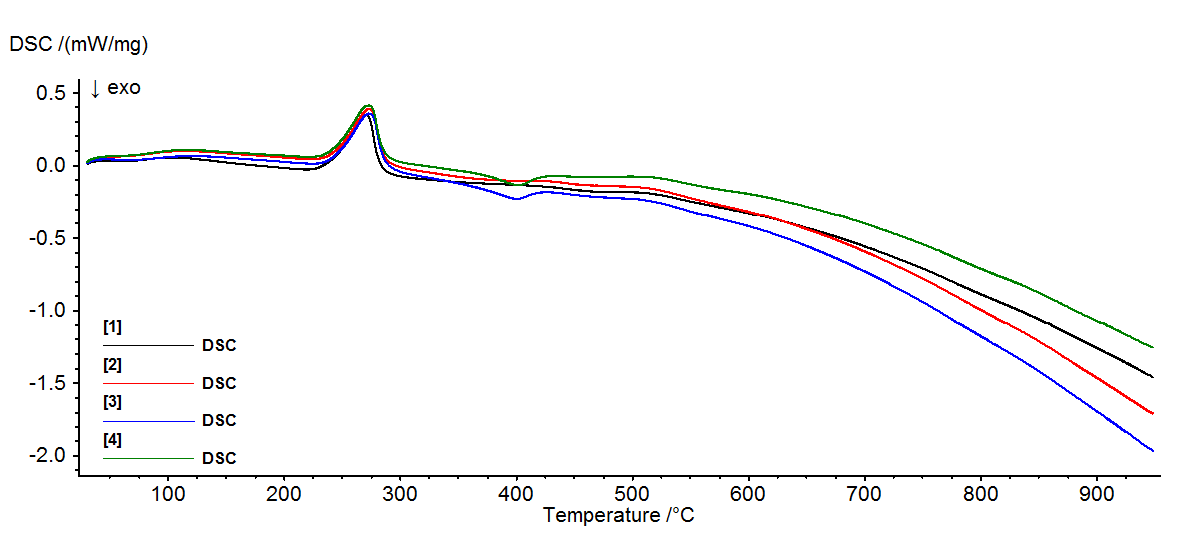
**

**Fig. 10.**

Fig. 6. Transmission and backscattering curves for Al_2_O_3_-PAM 14_30% system, pH 6, 15 ^o^C, C_PAM_ = 100 ppm.

Fig. 7. Transmission and backscattering curves for Al_2_O_3_ system without PAM, pH 9, 35 ^o^C.

Fig. 8. TG curves for alumina systems modified by polyacrylamide adsorbed at pH 3 and 35 ^o^C. [1] Al_2_O_3_; [2] Al_2_O_3_/PAM 11_5%; [3] Al_2_O_3_/PAM 14_20%; [4] Al_2_O_3_/PAM 14_30%.

Fig. 9. DTG curves for alumina systems modified by polyacrylamide adsorbed at pH 3 and 35 ^o^C. [1] Al_2_O_3_; [2] Al_2_O_3_/PAM 11_5%; [3] Al_2_O_3_/PAM 14_20%; [4] Al_2_O_3_/PAM 14_30%.

Fig. 10. DSC curves for alumina systems modified by polyacrylamide adsorbed at pH 3 and 35 ^o^C. [1] Al_2_O_3_; [2] Al_2_O_3_/PAM 11_5%; [3] Al_2_O_3_/PAM 14_20%; [4] Al_2_O_3_/PAM 14_30%.
